# Neuroendocrine Transformation as a Mechanism of Resistance to Targeted Lung Cancer Therapies: Emerging Mechanisms and Their Therapeutic Implications

**DOI:** 10.3390/cancers17020260

**Published:** 2025-01-15

**Authors:** Asim Joshi, Nivitha Bhaskar, Joel D. Pearson

**Affiliations:** 1 Department of Pharmacology & Therapeutics, Max Rady College of Medicine, University of Manitoba, Winnipeg, MB R3T 0T6, Canada; asim.joshi@umanitoba.ca (A.J.); nivitha.nivitha@umanitoba.ca (N.B.); 2 Paul Albrechtsen Research Institute, CancerCare Manitoba, Winnipeg, MB R3E 0V9, Canada; 3 Children’s Hospital Research Institute of Manitoba, Winnipeg, MB R3E 3P4, Canada

**Keywords:** lung cancer, small cell lung cancer (SCLC), histological transformation, neuroendocrine, drug resistance, plasticity, RB1, EGFR, tyrosine kinase inhibitors

## Abstract

Lung cancer is the leading cause of cancer-related deaths worldwide, highlighting a major clinical challenge. Lung cancer is broadly classified into two histologically distinct subtypes, termed small cell lung cancer (SCLC) or non-small cell lung cancer (NSCLC). Identification of various oncogenic drivers of NSCLC has facilitated the development of targeted therapies that have dramatically improved patient outcomes. However, acquired resistance to these targeted therapies is common, which ultimately results in patient relapse. Several on-target and off-target resistance mechanisms have been described for targeted therapies in NSCLC. One common off-target mechanism of resistance to these therapies is histological transformation of the initial NSCLC into SCLC, a highly aggressive form of lung cancer that exhibits neuroendocrine histology. This mechanism of resistance presents a significant clinical challenge, since there are very few treatments available for these relapsed patients. Although the phenomenon of NSCLC-to-SCLC transformation was described almost 20 years ago, only recently have we begun to understand the mechanisms underlying this therapy-driven response. These recent discoveries will be key to identifying novel biomarkers and therapeutic strategies to improve outcomes of patients that undergo NSCLC-to-SCLC transformation. Here, we highlight these recent advances and discuss the potential therapeutic strategies that they have uncovered to target this mechanism of resistance.

## Simple Summary

Over the last 20 years, the development of targeted therapies has dramatically improved the survival of patients with non-small cell lung cancer (NSCLC) that possess actionable driver mutations. However, most of these patients eventually relapse with drug-resistant disease. Understanding how these tumors develop resistance to targeted therapies will ultimately reveal novel therapeutic strategies to target these mechanisms of resistance and improve patient outcomes. While various mechanisms of resistance to these targeted therapies have been identified, one common mechanism is histological transformation of the initial NSCLC into small cell lung cancer (SCLC), a histologically distinct subtype of lung cancer. In this review, we highlight recent advances in our understanding of the mechanisms that drive NSCLC-to-SCLC transformation and how these discoveries have revealed potential therapies to target this mechanism of resistance.

## 1. Introduction

Lung cancer is broadly classified into two main histological subtypes, termed small cell lung cancer (SCLC, ~15% of cases) and non-small cell lung cancer (NSCLC, ~85% of cases) [1,2]. SCLC is described as a neuroendocrine (NE) cancer of the lung, whereas NSCLC is primarily of epithelial origin and is further divided into three main subtypes: adenocarcinoma (LUAD), squamous cell carcinoma and large cell carcinoma [2,3]. Various oncogenic driver mutations have been identified in NSCLC, with some of the most common being activating mutations in epidermal growth factor receptor (EGFR) and fusion proteins involving anaplastic lymphoma kinase (ALK) [2,3]. The discovery of these key oncogenic drivers has spurred the development of various targeted therapies, such as tyrosine kinase inhibitors (TKIs), which have provided significant survival benefits compared to traditional chemotherapy [4]. Unfortunately, the majority of tumors eventually develop resistance to these therapies through a variety of mechanisms [4]. One such mechanism is histological transformation of the initial NSCLC into SCLC, a process termed SCLC transformation (also referred to as small cell transformation or NE transformation) [5]. In these cases, relapsed tumors undergo significant molecular and histological changes and exhibit many classical characteristics of SCLC, such as high nuclear-to-cytoplasmic ratio, expression of NE markers and inactivation of the *RB1* and *p53* tumor suppressors [6,7,8]. Importantly, the transformed SCLC tumors retain the initial oncogenic mutation found in the original NSCLC, highlighting the likelihood of direct evolution of the initial NSCLC [6,7,8]. We refer to these instances of NSCLC-to-SCLC transformation as transformed SCLC, or T-SCLC, to distinguish them from SCLC that arises de novo. Unfortunately, there are few treatment options for patients with T-SCLC, which results in very poor outcomes for these patients [9,10,11,12]. Developing a better understanding of the mechanisms that drive NSCLC-to-SCLC transformation will ultimately identify novel treatment strategies to improve outcomes for these patients. However, we have only recently begun to understand these mechanisms and how they may inform clinical intervention. In this review, we highlight these recent advances and discuss the potential therapeutic opportunities that they have uncovered.

## 2. The Context of SCLC Transformation

### 2.1. EGFR-Mutant NSCLC

**Figure 1:**
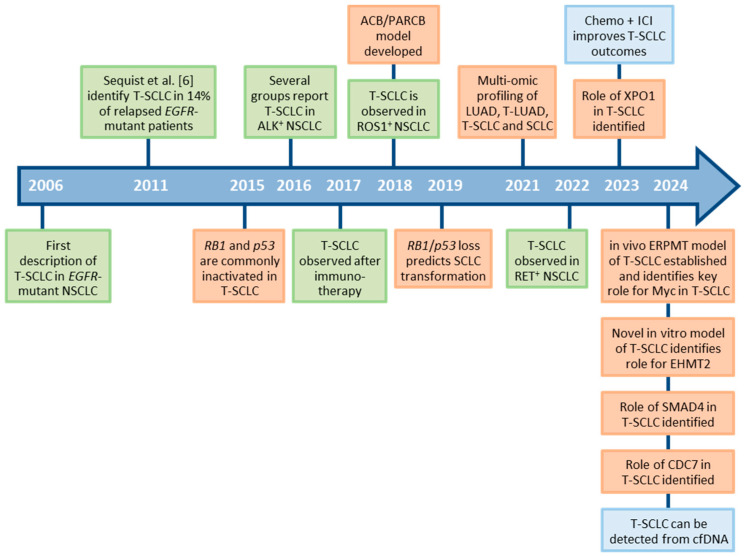
Timeline of discoveries related to SCLC transformation. Green boxes indicate observations in patients. Orange boxes indicate the development of new experimental model systems and mechanistic discoveries. Blue boxes indicate discoveries related to diagnosis and treatment of patients. T-SCLC: transformed small cell lung cancer; NSCLC: non-small cell lung cancer; LUAD: lung adenocarcinoma; ICI: immune checkpoint inhibitor; cfDNA: cell-free DNA.

SCLC transformation is best characterized in the context of *EGFR*-mutant NSCLC, where it has been reported that 3–14% of patients undergo SCLC transformation as a mechanism of resistance to EGFR TKIs [6,8,9,13]. The first case of NSCLC-to-SCLC transformation was reported in 2006 [14] (Figure 1). This case report described a 45-year-old female never smoker with advanced *EGFR*-mutant LUAD, who was initially treated with the EGFR TKI Erlotinib. After 18 months, her disease progressed, and treatment was switched to an alternative EGFR inhibitor, Gefitinib. Unfortunately, the patient was unresponsive to the new therapy, and at autopsy, metastases were found to be entirely SCLC with no residual LUAD [14]. Strikingly, these SCLC tumors possessed the same *EGFR* 18 bp deletion in exon 19 that was detected in the initial LUAD, suggesting the SCLC was clonally derived from the initial *EGFR*-mutant LUAD. Following this report, several subsequent case reports similarly described SCLC transformation following treatment of NSCLC patients with EGFR inhibitors [15,16,17,18]. However, the extent of SCLC transformation as a mechanism of resistance to EGFR inhibitors was not fully appreciated until a landmark study from Sequist et al., who profiled 37 NSCLC patients with treatment-resistant, *EGFR*-mutant NSCLC and identified five instances (14%) of SCLC transformation, highlighting this as a common mechanism of resistance [6].

### 2.2. Beyond EGFR-Mutant NSCLC

Although NSCLC-to-SCLC transformation is best characterized in patients with *EGFR*-mutant LUAD, SCLC transformation has also been described in various other contexts. Multiple reports have described SCLC transformation in patients with ALK-positive LUAD, primarily as a mechanism of resistance to the ALK TKI Alectinib [19,20,21,22,23,24,25]. Similarly, NSCLC in patients with ROS1 fusion proteins can transform into SCLC following targeted therapies [23,26,27]. Additionally, the first case of SCLC transformation from RET fusion-positive LUAD was recently identified in a patient with a KIF5B::RET fusion after treatment failure with the RET inhibitor, Pralsetinib [28]. Several other groups subsequently described SCLC transformation in RET-fusion NSCLC [23,29,30].

Beyond representing a mechanism of resistance to TKIs, SCLC transformation has also been described as a mechanism of resistance to immunotherapy in NSCLC. The first case of SCLC transformation following immunotherapy with Nivolumab was reported in 2017 [31]. Since then, several case reports have described instances of NSCLC-to-SCLC transformation following immunotherapy [32,33,34,35,36,37,38,39], which has occurred in both LUAD [33,34,36] and lung squamous cell carcinoma [32,35,37,38,39]. Given that SCLC is primarily considered an immune-cold tumor [40], SCLC transformation appears a logical route of resistance to immunotherapy. While most instances of T-SCLC following immunotherapy are based on individual case reports, one group noted SCLC transformation in two of the eight (25%) patients assessed after relapse [35]. Whether this frequency will hold true in larger cohorts remains to be determined, but these findings highlight the need to perform repeat biopsies on NSCLC patients following relapse on immunotherapy. Notably, none of the initial NSCLC tumors in these cases had known *EGFR* or *ALK* driver mutations, demonstrating that SCLC transformation can occur in contexts beyond those involving TKI therapies [32]. Consistent with this notion, others have noted SCLC transformation in NSCLC patients that lacked mutations in *EGFR* or *ALK* and that had never been treated with TKIs [9,41]. In one study, it was also argued that SCLC transformation is more common in NSCLC patients with wild type *EGFR/ALK* than those with mutant *EGFR/ALK* [9]. Future work with larger patient cohorts will be required to fully delineate the frequency of SCLC transformation in contexts outside of *EGFR* mutations, although these many studies suggest that this should be a focus, as it may be more common than initially believed.

## 3. Clinical Course of SCLC Transformation

Multiple studies have now followed the clinical course of patients undergoing NSCLC-to-SCLC transformation. In these studies, the average time to transformation in *EGFR*-mutant NSCLC ranges from 13–24 months after initial TKI treatment [9,10,11,12,13,41]. Studies exploring the timeline of transformation in non-*EGFR* mutant NSCLC are limited, but one study that compared *EGFR*-mutant to non-*EGFR* mutant NSCLC noted that non-mutant tumors took longer to transform to SCLC (26 months vs. 16 months for *EGFR*-mutant) [10]. Following transformation, patients with T-SCLC are treated with platinum/etoposide chemotherapy, the same standard-of-care used for de novo SCLC [10,12,42]. Much like de novo SCLC, T-SCLC patients often exhibit an initial response to platinum/etoposide, but this is typically followed by rapid clinical decline with mean overall survival ranging from 9–12 months post-transformation [9,10,11,12]. Interestingly, studies examining survival of T-SCLC derived from *EGFR*-mutant vs. *EGFR* wild type NSCLC have demonstrated differing outcomes, with one study showing reduced survival in the *EGFR* wild type group (6 vs. 10 months) [9], while the second study found no difference (9 vs. 10 months) [10]. Whether these discrepancies reflect distinct patient populations or are a consequence of small sample sizes remains to be determined, but regardless, these studies highlight the poor outcomes experienced by patients with T-SCLC, much like those of patients with de novo SCLC.

## 4. The Origins of T-SCLC

While the cellular origins of T-SCLC are not definitively known, there are two main theories as to how it arises. One possibility is that a population of SCLC clones may be present at a low frequency prior to initial treatment of the NSCLC but went undetected in the initial biopsy [7,43]. Following treatment, these cells possess a survival advantage and outgrow the NSCLC. While this may be the case in some instances, and indeed, mixed NSCLC/SCLC tumors have been observed at initial diagnosis in some patients [13], this theory seems at odds with the aggressive nature of SCLC, which is unlikely to remain dormant for extended periods. A more widely accepted theory is that NSCLC directly transforms to SCLC as a result of the selective pressure of treatment. Several retrospective studies support this theory, showing that T-SCLC arising from *EGFR*-mutant NSCLC still carries the original *EGFR* mutation [6,7,10,12]. In one specific case, an *EGFR*-mutant NSCLC patient (possessing an *EGFR* exon 19 deletion) was initially treated with multiple rounds of early-generation EGFR inhibitors but developed resistance via acquisition of the *EGFR* T790M gatekeeper mutation [44]. Following this, the patient was treated with the third-generation EGFR inhibitor, Osimertinib, and subsequently relapsed with T-SCLC [44]. Importantly, the T-SCLC contained both the initial *EGFR* exon 19 deletion and the acquired T790M mutation [44], strongly supporting a model of therapy-driven evolution. Alveolar type II cells are believed to be a primary cell of origin of LUAD, while SCLC primarily arises from pulmonary NE cells [45]. Interestingly, alveolar type II cells can also give rise to SCLC at a low frequency [46], further supporting the theory that T-SCLC can evolve directly from LUAD.

## 5. Preclinical Models of SCLC Transformation

**Figure 2:**
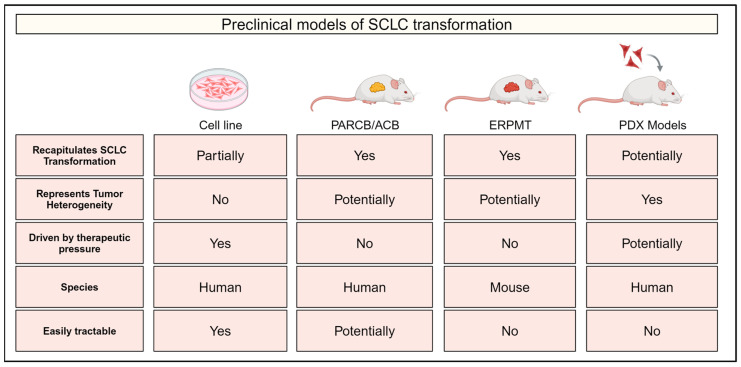
Experimental models of small cell lung cancer (SCLC) transformation. Comparison of experimental models of SCLC transformation indicating whether each model recapitulates SCLC evolution/transformation, tumor heterogeneity and selection driven by therapeutic pressure. Information is also provided on the species of each model and whether they represent an easily tractable system. PARCB/ACB: genetically engineered human xenograft model using manipulations in p53, AKT, RB1, c-MYC and BCL2. ERPMT: genetically engineered mouse model possessing transgenes for *EGFR^L858R^*, *Myc^T58A^* and *tdTomato* and floxed alleles of *p53* and *Rb1*. PDX: patient-derived xenograft.

Recent advances in our understanding of SCLC transformation have been facilitated by the development of several novel models. These have spanned from in vitro cell line models to xenograft systems to genetically engineered mouse models (Figure 2). In this section, we highlight these different systems and how they have been utilized to deduce mechanisms of SCLC transformation, although we save the details of these mechanisms for a later section.

### 5.1. Cell Line Models

The establishment of in vitro models of therapy-driven NSCLC-to-SCLC transformation has been particularly challenging, potentially due to specific conditions found in vivo that cannot be easily recapitulated in vitro or due to limitations of long-established cell lines that do not represent the heterogeneity of human tumors [47]. However, a recent study by Yang et al. demonstrated that *EGFR*-mutant NSCLC lines can convert to an NE-like phenotype as a mechanism of resistance to the EGFR inhibitor, Erlotinib [48]. Following a classic dose-escalation regimen with Erlotinib, the authors established resistant populations from several NSCLC lines, which were then clonally selected to identify clones that exhibited a more NE-like phenotype, as the initial resistant populations exhibited significant heterogeneity [48]. Resistant NE-like sub-clones derived from four different NSCLC lines exhibited downregulation of RB1 and EGFR protein; upregulation of classic NE markers, such as CHGA; and increased sensitivity to chemotherapeutic agents used to treated SCLC, suggesting transition to an SCLC-like state [48]. However, unlike T-SCLC, these lines did not acquire *RB1* gene mutations or loss, with protein downregulation likely a result of epigenetic mechanisms [48]. Furthermore, the cell lines exhibited an adherent morphology [48], which is distinct from the classic non-adherent morphology of SCLC and most other NE cell lines [49]. Thus, these findings suggest that while this model recapitulates some aspects of SCLC transformation, the cells may not undergo full transformation. This approach may be complicated by the fact that standard cell culture conditions naturally select for adherent cells. Regardless, this in vitro model provides the unique advantage of being a system that can be readily manipulated for mechanistic studies. Furthermore, it is also driven by therapeutic pressure, which more closely mirrors patient treatment, unlike many other models discussed below that rely on genetic approaches to drive transformation.

### 5.2. The ACB/PARCB Model

The “ACB/PARCB” model is a genetically modified human xenograft model developed by Park et al. to recapitulate NE transformation (e.g., SCLC transformation) from various lineages including lung, prostate and bladder [50,51,52]. In this model, normal human epithelial cells from lung or other tissues are transduced with a cocktail of oncogenes and then xenografted into immunodeficient mice, where they form tumors that histologically resemble either adenocarcinoma or small cell NE cancer (e.g., SCLC). When cells are engineered to over-express MYC, BCL2 and activated AKT (ACB genes, for AKT, c-MYC and BCL2), they transform to adenocarcinoma. In contrast, over-expression of these three genes along with knockdown of RB1 and over-expression of dominant-negative p53 (PARCB genes; ACB genes plus p53 and RB1 inactivation) drives temporal evolution from adenocarcinoma to an NE tumor that histologically and transcriptionally matches primary NE tumors, such as SCLC [50,51]. This observation elegantly underscores the pivotal role of p53 and RB1 loss in SCLC transformation. In addition, using integrated analysis of the epigenome and transcriptome, the authors revealed that RB1 and p53 inactivation drives genome-wide epigenetic reprogramming accompanied by major changes in chromatin accessibility [50]. This epigenetic reprogramming included silencing of p53-binding sites and opening of many sites bound by known NE lineage factors (e.g., ASCL1) [50], thus providing insights into putative mechanisms of transformation.

### 5.3. The Genetically Engineered ERPMT Mouse Model

The genetically engineered ERPMT (EGFR^L858R^; Rb1^floxed^; p53^floxed^; Myc^T58A^; tdTomato) mouse model developed by Gardner et al. is another robust system that truly mimics the lineage conversion process [53]. This model builds on the standard SCLC mouse model of conditional *Rb1* and *p53* knockout [46,54] by including over-expression of stabilized Myc (Myc^T58A^) and mutant *EGFR* (L858R mutation found in patients) [53]. Importantly, the mutant *EGFR* is under the control of a doxycycline-inducible promoter, allowing its expression to be turned “on” or “off” through the addition or removal of doxycycline [53]. The authors demonstrated that when mutant *EGFR* was “on”, mice specifically developed LUAD from alveolar type II cells, whereas mice developed SCLC from pulmonary neuroendocrine cells when EGFR was “off”. This demonstrates striking lineage-specific susceptibility to distinct oncogenic insults [53]. Importantly, if mutant *EGFR* expression was turned “off” (via removal of doxycycline) after LUAD was allowed to establish, the LUAD underwent histological transformation to SCLC [53]. Temporal single cell transcriptomic analysis of these tumors demonstrated that once EGFR was turned “off”, cells first dedifferentiated to a high plasticity, lineage-negative stem/progenitor-like state driven by high Myc activity. This state then appeared to form a “bottleneck” where some cells were able to reprogram and emerge as T-SCLC [53]. To date, this model provides the most comprehensive temporal analysis of tumor cells undergoing histological transformation and highlights key roles for Myc, Rb1 and the PI3K/Akt pathway in transformation, as these pathways are commonly altered in human T-SCLC.

### 5.4. Patient-Derived Xenograft Models

Apart from the engineered human and mouse models described above, patient-derived xenograft (PDX) models have emerged as complementary tools to study histological transformation. While these systems may be less amenable to manipulation compared to cell line models, they more accurately recapitulate the heterogeneity of human tumors. For example, Quintanal-Villalonga et al. established a PDX model from an *EGFR*-mutant patient with combined LUAD and SCLC histology and demonstrated that the PDX retained both the LUAD and SCLC features of the original tumor [55]. The authors were then able to utilize this model to demonstrate a key role for PI3K/AKT activity in T-SCLC [55] and have subsequently used this unique model to dissect the role of several other pathways in T-SCLC [56,57]. Another study utilized a PDX model derived from an *EGFR/p53/RB1*-altered LUAD patient. Interestingly, knockdown of EGFR in this model resulted in induction of select neuroendocrine markers, suggesting its potential to transform into SCLC [58]. This highlights the broad utility of these patient-derived models for studying SCLC transformation. Together, these and other complementary in vitro and in vivo models of histological transformation have provided the platforms to dissect the complex mechanisms underlying SCLC transformation.

## 6. Mechanisms Underlying NSCLC-to-SCLC Transformation

**Figure 3:**
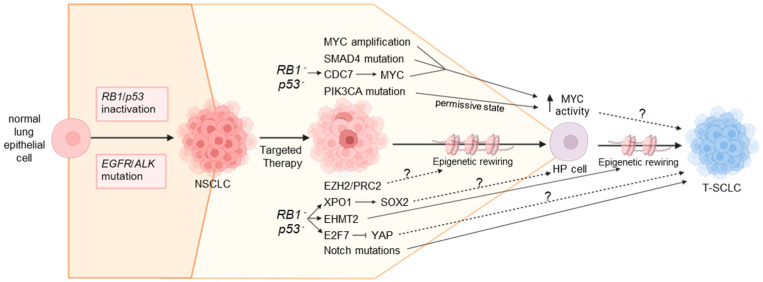
Mechanisms underlying SCLC transformation. SCLC transformation is likely a step-wise process driven by multiple genomic alterations, transcriptional changes and epigenetic rewiring. Inactivation of the *RB1* and *p53* tumor suppressors is a key early event that predisposes NSCLC to transformation and likely sits upstream of several key pathways involved, including upregulation of CDC7, XPO1 and EHMT2 and downregulation of YAP/TAZ. Several of these events converge on MYC, which drives de-differentiation to a high plasticity (HP) stem-like state. Cells then differentiate from this HP state towards a neuroendocrine phenotype indicative of SCLC. We speculate that this latter step is facilitated by YAP/TAZ downregulation, Notch pathway mutations and epigenetic rewiring driven by EHMT2 upregulation, although multiple other pathways are likely involved. T-SCLC: transformed small cell lung cancer; NSCLC: non-small cell lung cancer; HP: high plasticity. Solid arrows indicate experimentally validated mechanisms; dotted arrows indicate speculative mechanisms that have yet to be experimentally confirmed.

While we still have much to learn about the underlying mechanisms that drive NSCLC-to-SCLC transformation, in recent years, the field has made major gains in our understanding of these mechanisms (Figure 3). Thus far, much of this work has been inspired by genomic, transcriptomic and epigenomic studies comparing paired tumor samples pre- and post-transformation or by comparing adjacent regions of tumors that exhibit mixed NSCLC/SCLC histology. This work has repeatedly demonstrated that there are no consistent mutational differences between pre-transformed NSCLC and their matched T-SCLC; however, there are multiple recurrent alterations found in both populations that are associated with transformation [9,11,23,55]. This suggests that these genomic alterations likely play an important role in SCLC transformation but that additional mechanisms, such as transcriptional and epigenetic reprogramming, are ultimately necessary to drive transformation. Here, we discuss the genomic, transcriptomic and epigenomic mechanisms that likely play important roles in driving NSCLC-to-SCLC transformation.

### 6.1. Genomic Alterations Influencing SCLC Transformation

While transformation to SCLC is not commonly associated with acquisition of new genomic alterations [9,11,23,55], there are several recurrent mutations associated with T-SCLC that likely play a key role in transformation and may predispose certain tumors to transformation. These include inactivation of the *RB1* and *p53* tumor suppressors [7,9,11,12,13,23,43,55]; amplification of *MYC* [11,13,23,43], *CCNE1* [11,13,23] and *NKX2.1* [9,13,23]; and mutations in members of the PI3K/AKT pathway (primarily *PIK3CA*) [6,7,9,11,12,13,23,43,55], Notch pathway [9,12,13,44,55] and multiple epigenetic regulators, such as members of the KMT2 family [9,55]. Many of these alterations are also commonly observed in de novo SCLC [23,55,59,60], suggesting common molecular underpinnings shared by these two forms of SCLC. Below, we discuss several common mutations associated with SCLC transformation and how these may be involved in the process of SCLC transformation.

#### 6.1.1. EGFR Exon19 Deletions

SCLC transformation is most commonly described in patients with *EGFR*-mutant NSCLC. Interestingly, several groups reported over-representation of *EGFR* exon 19 deletions in cases of T-SCLC compared to *EGFR* L858R mutations. Several different *EGFR* mutations are observed in NSCLC, with exon 19 deletion and L858R point mutation being the most common. In the overall population of *EGFR*-mutant NSCLC patients, these two alterations are observed at roughly equal proportions, although this can vary depending on stage and ethnicity [61,62,63]. Interestingly, in T-SCLC that arises from *EGFR*-mutant NSCLC, exon 19 deletions are present in 64–78% of patients, while L858R mutations are present in only 11–25% of patients [9,11,12,23,43,64]. Why there is enrichment of *EGFR* exon 19 deletions in T-SCLC is unknown but is very intriguing. It is possible this reflects a true propensity of *EGFR* exon 19 deletion to facilitate conversion through yet unknown mechanisms. Alternatively, this may instead suggest that specific patient demographics are more likely to undergo SCLC transformation. For example, exon 19 mutations are more common than L858R mutations in patients of Asian descent and in non-smokers [62]. Deducing the underlying reasons for over-representation of *EGFR* exon 19 deletions in T-SCLC warrants further investigation and will require careful consideration of specific patient populations.

#### 6.1.2. RB1 and p53 Inactivation

The first genomic alterations described in T-SCLC patient samples were biallelic inactivation of the *RB1* and *p53* tumor suppressors, which are observed in the majority of cases [7,9,11,12,13,23,43,44,55]. This matches de novo SCLC where *RB1* and *p53* are also commonly inactivated [23,59,60,65,66]. In the majority of T-SCLC patients, *p53* and *RB1* inactivation is observed in the pre-transformed NSCLC, and combined loss of both genes is currently the strongest predictor of SCLC transformation in *EGFR*-mutant NSCLC patients [12,13,43,55]. This also suggests that *RB1*/*p53* inactivation is likely an important early event underlying SCLC transformation, although it is important to note that the majority of *EGFR/RB1/p53*-mutant NSCLC tumors do not undergo SCLC transformation [13], highlighting the importance of other molecular drivers. In support of a key role for *RB1/p53* inactivation in SCLC transformation, *Rb1* loss is required for NSCLC-to-SCLC conversion in the ERPMT mouse model [53] and ACB/PARCB xenograft model [50]. How *RB1* and *p53* loss influences SCLC transformation is likely multifactorial, as it appears to be upstream of several pathways discussed later, some of which are likely dependent on the canonical RB1/E2F pathway. Interestingly, in the ACB/PARCB xenograft model, inactivation of both RB1 and p53 drives wide-scale chromatin remodeling associated with NE transformation [50]. This is consistent with the known roles of RB1 and p53 in regulating chromatin architecture [67,68] and suggests this may represent a key function of RB1/p53 in this context.

#### 6.1.3. MYC Amplification

Amplification of the *MYC* proto-oncogene has been associated with SCLC transformation in multiple studies, being identified in 8–14% of patients [11,13,23]. This observation is consistent with de novo SCLC, where MYC family members (*MYC*, *MYCN* and *MYCL*) are commonly amplified [23,59,60,66,69]. As with other mutations associated with SCLC transformation, *MYC* amplifications are often present in the pre-transformed NSCLC tumor, suggesting this may predispose certain patients to SCLC transformation. Additionally, high MYC activity correlates with SCLC transformation in multiple experimental models [53,57], and MYC over-expression is a key component of the oncogenic cocktail that drives NE conversion in the ACB/PARCB xenograft model [50,51], further implicating MYC in SCLC transformation.

Despite multiple studies implicating MYC in SCLC transformation, it was not until recently that a key mechanism underlying MYC function in this context was revealed. In their genetically engineered ERPMT mouse model of SCLC transformation, Gardner et al. elegantly demonstrated that Myc over-expression cooperates with *Rb1* and *p53* loss to drive reprogramming of *EGFR*-mutant LUAD into a highly undifferentiated, stem/progenitor-like state following blockade of EGFR activity [53]. Following de-differentiation, these progenitor-like cells reprogram and emerge as aggressive SCLC characterized by high Myc and Sox2 activity [53]. In this mouse model, Myc over-expression was required for efficient SCLC transformation, since tumor penetrance dropped by 50% in mice lacking Myc over-expression [53]. Furthermore, in mice that lacked Myc over-expression, tumors exhibited a mixture of LUAD and SCLC histology, in contrast to Myc over-expressing mice that exclusively developed SCLC [53]. It was further demonstrated that most cells of the mouse lung, including alveolar type II cells (the presumed cell of origin of LUAD), are resistant to transformation by Myc over-expression alone and instead undergo cell death [53]. In contrast, pulmonary NE cells (a presumed cell of origin of SCLC) are permissive to transformation by Myc [53]. This highlights a unique cellular context specific to SCLC that is permissive to high Myc activity and identifies Myc as a key driver of SCLC transformation. Interestingly, however, inactivation of *Pten* was sufficient to permit transformation of alveolar cells by Myc [53]. Furthermore, knockout of *Pten* allowed for the genesis of SCLC from alveolar cells in the context of Myc over-expression and *Rb1/p53* knockout [53], which may partially explain the high prevalence of PI3K/AKT pathway mutations in T-SCLC patients [6,7,9,11,12,13,23,43,55]. An intriguing question that arises from this work is why T-SCLC is primarily associated with amplification of *MYC* [11,13,23], whereas all MYC family members (*MYC*, *MYCN* and *MYCL*) are routinely amplified in de novo SCLC [23,59,60,66,69]. Perhaps this is merely a reflection of the small size of T-SCLC cohorts or, more interestingly, it may reflect a unique function of MYC in driving cellular reprogramming that is not shared by its related family members.

#### 6.1.4. Notch Pathway Mutations

Mutations and downregulation of various Notch pathway members have been associated with T-SCLC in several patient cohorts [9,23,44,55], and transcriptomic profiling of patient tumors demonstrated that Notch activity is reduced in T-SCLC [55]. Furthermore, in patient samples, Notch activity is reduced in LUAD that eventually undergoes SCLC transformation (i.e., the pre-transformed NSCLC) compared to LUAD that never transforms to SCLC [55]. Similar results were observed in the ERPMT mouse model of SCLC transformation [53], highlighting reduced Notch signaling as a conserved feature of SCLC transformation. While the role of Notch mutations and reduced Notch signaling in SCLC transformation has not been functionally assessed, it is conceivable that it plays an important role. For example, Notch activation inhibits NE differentiation in SCLC and various other contexts, at least partially through downregulating ASCL1, a master regulator of NE differentiation [70,71,72,73,74].

#### 6.1.5. SMAD4 Mutations

In a cohort of eight matched pre- and post-transformation SCLC patient samples, *SMAD4*-inactivating mutations were identified in two patients (25% of samples) [9]. In one case, *SMAD4* mutation was present in the pre-transformed NSCLC sample, and in the other, it was gained during transformation, which was consistent with previous reports showing acquisition of rare *SMAD4* mutations in patients with T-SCLC [12,75]. The authors further demonstrated that SMAD4 expression is reduced in T-SCLC relative to pre-transformed patient tumors, even in tumors with wild type *SMAD4* [9]. Importantly, knockout of *SMAD4* in *p53*-mutant NSCLC cell lines induced expression of key NE markers, such as ASCL1, which was greatly enhanced when *RB1* was also knocked out [9]. Knockout of both *SMAD4* and *RB1* in these lines also conferred resistance to EGFR inhibitors [9]. Mechanistically, it was proposed that SMAD4 and MYC compete for binding to the MYC co-factor MAX, and that SMAD4/MAX complexes silence ASCL1 transcription, whereas MYC/MAX induce ASCL1 transcription [9]. Thus, mutation or downregulation of SMAD4 permits the formation of MYC/MAX complexes that drive transcription of ASCL1 and SCLC transformation. ASCL1 is a master regulator of NE differentiation and a marker for SCLC; however, recent work defined different SCLC molecular subtypes based on the expression of lineage-determining transcriptions factors and demonstrated that subsets of SCLC do not express ASCL1 and are instead driven by other key transcription factors, such as NEUROD1 or POU2F3 [76]. Given that NSCLC can transform into multiple different SCLC subtypes [55,77], it will be interesting to determine if this role for SMAD4 is restricted to ASCL1-positive T-SCLC or if it is also relevant to other subtypes.

### 6.2. Transcriptional Mechanisms Influencing SCLC Transformation

The lack of recurrent mutational differences between pre- and post-transformed SCLC [9,11,23,55] has led to a widespread belief that transcriptional and epigenetic mechanisms likely play a key role in driving SCLC transformation. However, it is only recently that comprehensive transcriptomic datasets have been generated comparing pre- and post-transformed SCLC, which have begun to uncover some of these mechanisms. In a seminal study, Quintanal-Villalonga et al. performed multi-omic profiling of primary human tumors from pre- and post-transformed SCLC as well as several tumors that exhibited mixed LUAD/SCLC histology, which were micro-dissected to separate NSCLC and SCLC regions [55]. These mixed-histology and pre-transformed LUAD are referred to as “T-LUAD” to distinguish them from LUAD that never underwent histological transformation. T-LUAD and T-SCLC tumors were then compared to LUAD tumors that never transformed and de novo SCLC, which are termed LUAD and SCLC, respectively [55]. Transcriptional profiling of these patient samples demonstrated widespread transcriptional changes that clearly distinguished LUAD, T-LUAD, T-SCLC and SCLC tumors, with tumors showing a progressive change from LUAD → T-LUAD → T-SCLC → SCLC [55]. These striking findings support the notion that certain NSCLC tumors are transcriptionally primed to undergo SCLC transformation. Further analysis of transcriptional differences between T-LUAD and T-SCLC revealed increased expression of cell cycle, DNA repair, chromatin remodeling, WNT signaling and PR2C target genes in T-SCLC, along with decreased expression of negative regulators of PI3K/AKT signaling, immune-related genes and RTK signaling [55]. Importantly, many of these altered pathways are consistent with the mutational signatures shared between T-LUAD and T-SCLC (e.g., mutations in PI3K/AKT, WNT and epigenetic regulators) [55], suggesting these mutations may prime tumors for SCLC transformation. This work set the stage for subsequent studies that have identified key mechanisms driving SCLC transformation.

#### 6.2.1. Exportin 1 Upregulation

Exportin 1 (XPO1) is a protein transporter responsible for nuclear-cytoplasmic export of various cargo proteins [78] that was previously identified as a therapeutic vulnerability in SCLC and other NE cancers [49,79,80]. Using their T-LUAD and T-SCLC datasets [55], Quintanal-Villalonga et al. found that XPO1 was upregulated in T-SCLC tumors relative to T-LUAD, with similar trends observed in prostate cancer models of NE transformation, suggesting that XPO1 may facilitate NSCLC-to-SCLC transformation [56]. XPO1 was upregulated following *RB1/p53* inactivation in human lung and prostate adenocarcinoma cell lines, which was likely through transcriptional regulation via p53 and E2F1, placing XPO1 directly downstream of *RB1/p53* loss [56]. The authors went on to demonstrate that inhibition of XPO1 using Selinexor, a clinically-relevant XPO1 inhibitor, prevented NE transformation and prolonged the effects of targeted therapies in both prostate and lung cancer xenograft models of NE transformation [56]. Mechanistically, it was demonstrated that XPO1 mediated induction of SOX2 [56], a key factor required for NE transformation in prostate cancer [81]. Whether SOX2 has a similar role in SCLC transformation is unknown, but given the many parallels between NE transformation in the lung and prostate and the critical role of SOX2 in controlling NE features of SCLC [82], this is entirely possible. Of note, high Sox2 activity was associated with SCLC transformation in the ERPMT mouse model [53], further suggesting a role for SOX2 in SCLC transformation.

#### 6.2.2. CDC7 Upregulation

Using a CRISPR dropout screen in cells cultured from a T-SCLC PDX, Quintanal-Villalonga et al. further identified the cell cycle regulator, CDC7, as an essential gene in T-SCLC [57]. Mirroring their previous results on XPO1, they demonstrated that CDC7 expression increased progressively from LUAD → T-LUAD → T-SCLC → SCLC in patient tumors, with CDC7 transcription induced following *RB1* and *p53* loss in adenocarcinoma cell lines, likely through direct regulation by p53 and E2F1 [57]. Importantly, dependency on CDC7 was induced following *RB1* and *p53* loss, and the CDC7 inhibitor, Simurosertib, synergized with EGFR and androgen receptor inhibitors in xenograft models of LUAD and prostate cancer NE transformation, respectively [57]. Mechanistically, CDC7 promoted protein stabilization and expression of MYC [57], a key mediator of SCLC transformation [53]. Importantly, forced expression of stabilized MYC (MYC^T58A^) rescued NE transformation in prostate cancer models following treatment with a CDC7 inhibitor [57], implicating MYC as a key downstream effector of CDC7 during NE transformation.

#### 6.2.3. FGF9 Upregulation

In a separate patient cohort, comparison of pre- and post-transformation SCLC tumors also identified upregulation of *FGF9* in four out of six cases of T-SCLC, with the other two cases exhibiting FGF9 expression in both the pre- and post-transformation tumors [83]. The authors further found that over-expression of FGF9 in a mouse LUAD cell line resulted in upregulation of SCLC markers and conversion to a mixed adherent/suspension morphology in vitro, consistent with SCLC transformation [83]. Furthermore, over-expression of FGF9 along with RB1 knockdown in a human *EGFR/p53*-mutant LUAD cell line resulted in induction of the NE marker ASCL1 following treatment with an EGFR TKI [83]. Similar results were found in an *EGFR* wild type, *RB1/p53*-mutant cell line xenograft, although in both instances, transformation was observed with only one of three lines tested [83]. Thus, FGF9 upregulation may play a role in driving SCLC transformation in certain contexts.

#### 6.2.4. YAP and TAZ Downregulation

The transcriptional co-activator YAP (YAP1) and its paralog TAZ (WWTR1) are downstream effectors of the Hippo signaling pathway and have well-characterized oncogenic roles in many solid cancers, including NSCLC [84,85]. However, the majority of SCLC lack expression of YAP and TAZ, with the exception of rare YAP-positive (e.g., SCLC-Y) cases [49,76,86,87,88,89]. In SCLC cell lines, loss of *RB1* results in E2F7 upregulation and epigenetic silencing of YAP [90], which is mediated by the RCOR-HDAC1/2-KDM1A co-repressor (CoREST) complex, although the requirement of KDM1A may vary between different cell lines [90,91]. Importantly, silencing of YAP/TAZ is required for proliferation and metastasis of SCLC and other NE cancers in vitro and in vivo [49,90,91,92,93,94,95], starkly contrasting the oncogenic role of YAP/TAZ in most other solid cancers, including NSCLC [84,85]. In the context of SCLC transformation, YAP is downregulated in most T-SCLC patient samples [48,55] as well as in human cell line and xenograft models of SCLC transformation [48,49]. Whether TAZ was also downregulated in patient samples was not assessed, but TAZ was downregulated in the ACB/PARCB xenograft model [49]. Given that YAP counteracts the NE fate of normal and transformed lung cells [70,92,93], likely through a NOTCH/REST axis [70,92], it is likely that YAP/TAZ silencing facilitates SCLC transformation, although whether this is an early or late event is currently unknown.

### 6.3. Epigenetic Remodeling During SCLC Transformation

Consistent with the transcriptional rewiring observed during NSCLC-to-SCLC transformation, tumor cells also undergo significant epigenetic reprogramming during transformation. In their collection of never transformed LUAD, T-LUAD, T-SCLC and de novo SCLC tumor samples, Quintanal-Villalonga et al. demonstrated progressive changes in DNA methylation as tumors transition from LUAD to T-LUAD to T-SCLC [55]. Despite this, T-SCLC methylation patterns more closely resembled LUAD than de novo SCLC, suggesting that T-SCLC retains certain epigenetic imprints of the initial tumor from which they evolved [55]. Importantly, however, differential methylation patterns explained many of the genes differentially expressed between T-LUAD and T-SCLC tumors, and motifs for key SCLC transcription factors, such as ASCL1 and NEUROD1, became hypomethylated as tumors transitioned to T-SCLC [55]. Furthermore, activator protein-1 (AP-1) sites were one of the top hypermethylated motifs in T-SCLC tumors [55], which is of particular note, since AP-1 proteins are critical partners of YAP/TAZ [96,97], which, as discussed above, are silenced during SCLC transformation [48,49,55].

**Figure 4:**
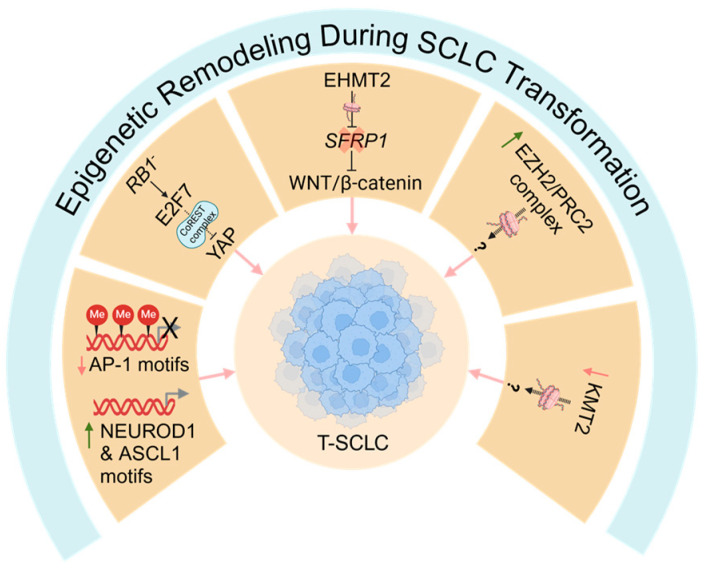
Epigenetic remodeling during SCLC transformation. Several epigenetic mechanisms likely contribute to SCLC transformation. SCLC transformation is associated with epigenetic silencing of activator protein-1 (AP-1) binding sites/motifs and activation of sites for classic neuroendocrine transcription factors, such as NEURDO1 and ASCL1. Furthermore, the anti-neuroendocrine transcription factors YAP and TAZ, which are partners of AP1, are epigenetically silenced, likely downstream of an RB1/E2F7/CoREST axis. The histone methyltransferase EHMT2 is upregulated and contributes to SCLC transformation, potentially by silencing SFRP1, a negative regulator of the WNT/β-catenin pathway. Furthermore, transformation is associated with increased EZH2/PRC2 activity and mutations in the KMT2 family of histone methyltransferases, although a functional role for these pathways in SCLC transformation is unclear. T-SCLC: transformed small cell lung cancer; AP-1: activator protein-1; PRC2: polycomb repressive complex 2. Red down arrows indicate genes/motifs downregulated during SCLC transformation; green up arrows indicate genes/motifs upregulated during SCLC transformation; question marks (?) indicate speculative mechanisms that have not yet been experimentally confirmed.

The above findings are consistent with, although slightly distinct from, a recent study that performed thorough epigenomic profiling of SCLC, T-SCLC and LUAD PDXs by assessing DNA methylation, histone modifications (H3K27Ac, H3K27me3 and H3K4me3) and chromatin accessibility (ATAC-Seq) [98]. This work demonstrated high overlap between de novo and T-SCLC, with patterns that were distinct from LUAD [98], suggesting that T-SCLC undergoes epigenetic rewiring as it evolves from LUAD. Similar findings in the ACB/PARB xenograft model of NE transformation demonstrated widespread changes in chromatin accessibility following NE transformation [50]. Furthermore, these NE-transformed xenografts showed hypo-accessibility of motifs associated with key NE transcription factors, such as ASCL1 and NEUROD1, and silencing of AP-1 motifs [50], consistent with the observations of Quintanal-Villalonga et al. [55]. Thus, several studies demonstrate that SCLC transformation is associated with significant epigenetic remodeling, particularly at key NE genes, which highlights a role for epigenetic regulators in SCLC transformation (Figure 4).

#### Mediators of Epigenetic Remodeling During SCLC Transformation

Transformation to SCLC is associated with mutations in several epigenetic regulators, such as members of the KMT2 family of lysine methyltransferases [9,55], and upregulation of various epigenetic and chromatin remodeling enzymes, such as EZH2 and other members of polycomb repressive complex 2 (PRC2) [55]. In a PDX model exhibiting mixed LUAD/SCLC histology, treatment with an EZH2 inhibitor did not overly impact the survival or histology of these tumors [55]. Furthermore, in an in vitro cell line model of SCLC transformation, inhibition of EZH2 did not revert the NE phenotype of transformed cell lines [48]. Together, these studies suggest that EZH2/PRC2 is not required to maintain the NE phenotype of T-SCLC, although, they do not rule out a role for EZH2 in earlier stages of transformation. Given that EZH2 is a critical mediator of therapy-induced NE differentiation in prostate cancer [99], it is possible that it may play a role at specific stages of SCLC transformation.

Recent work has, however, implicated the histone lysine methyltransferase, EHMT2 (G9a), in epigenetic remodeling during SCLC transformation. In their cell line model of SCLC transformation, Yang et al. found that EHMT2 was strongly upregulated in in vitro-derived T-SCLC as well as in T-SCLC patient tumors [48]. In these T-SCLC cell line models, genetic knockdown or pharmacological inhibition of EHMT2 reversed the NE phenotype of resistant cells and resensitized cells to EGFR inhibitors [48], implicating EHMT2 as a key driver of NE conversion in this context. Mechanistically, EHMT2 promoted activation of WNT/β-catenin signaling in T-SCLC cell lines through epigenetic silencing of *SFRP1*, a negative regulator of the WNT/β-catenin pathway [48]. Furthermore, pharmacological inhibition of WNT/β-catenin signaling reversed the NE phenotype of these derived T-SCLC cell lines [48], highlighting a key EHMT2-WNT/β-catenin axis during SCLC transformation. Interestingly, previous genomic profiling identified mutations in several WNT pathway members and increased WNT/β-catenin signaling in T-SCLC patient tumors, although in that work, combined treatment of a mixed LUAD/SCLC PDX with a WNT and EGFR inhibitor did not improve survival beyond an EGFR inhibitor alone [55], suggesting that WNT signaling was not critical in that context. Thus, the role of WNT signaling may be context-dependent in T-SCLC, although further work is required to determine this.

## 7. Timing of Events That Drive SCLC Transformation

Transformation of NSCLC to SCLC is undoubtedly a progressive process; however, we still have limited understanding of the temporal changes that occur during transformation and the mechanisms that drive these changes. The recent development of models that recapitulate SCLC transformation has begun to shed light on the potential timing of events that drive NSCLC-to-SCLC evolution, although much is still left up to speculation (Figure 3). Inactivation of *RB1* and *p53* in pre-transformed NSCLC is strongly associated with SCLC transformation [13] and is upstream of several key pathways [48,56,57], suggesting *RB1* and *p53* inactivation is likely an early event during transformation. Elegant work by Gardner et al. using their ERPMT mouse model of transformation identified an undifferentiated, high-plasticity stem-like state driven by high Myc activity that precedes SCLC transformation and forms a “bottleneck” point on the pathway of NSCLC-to-SCLC evolution [53]. This therefore suggests that *MYC* amplification and other events that impinge on MYC activity, such as *SMAD4* mutation [9] or CDC7 upregulation [57], may also represent early events during transformation that drive initial de-differentiation of LUAD prior to SCLC conversion. Activation of PI3K/AKT signaling (e.g., through *PIK3CA* or *PTEN* mutation) may also be important in these early stages to allow a cellular state that is permissive to high MYC activity [53], potentially by protecting against MYC-induced apoptosis [100]. These highly undifferentiated cells are also characterized by high expression of Sox2 targets [53]. XPO1 is upregulated downstream of *RB1/p53* inactivation and was shown to play a key role in T-SCLC, potentially via the upregulation of SOX2 [56], suggesting that induction of XPO1 may also contribute to formation of this high-plasticity intermediate state by driving SOX2 expression.

Following de-differentiation to this high-plasticity state, tumor cells likely undergo epigenetic and transcriptional reprogramming that drives differentiation towards the NE lineage and, ultimately, SCLC evolution. In the ERPMT mouse model, T-SCLC is also characterized by high Myc activity, which is increased beyond that of the high plasticity stem-like state [53]. This suggests a key role for Myc in latter stages of transformation as well as the initial role in driving de-differentiation. While other cues that drive NE differentiation from the progenitor-like bottleneck state are unknown, one could speculate that downregulation of YAP/TAZ and inactivation of Notch signaling may be involved given the well-known roles of these pathways in counteracting NE differentiation [70,71,72,73,74,92,93]. Whether these events may also be involved earlier along this trajectory of evolution are unknown, but YAP/TAZ have well-characterized roles in stem cell maintenance [101,102,103]. Thus, one could speculate that early expression of YAP/TAZ may be required for generation of the undifferentiated stem-like cells during SCLC transformation, and that, perhaps, YAP/TAZ downregulation is a key event that drives progression form this bottleneck towards an NE lineage. Various epigenetic factors also likely play key roles during SCLC transformation (Figure 4), although the timing of such events is unknown. EHMT2 was demonstrated to promote SCLC transformation from LUAD lines in vitro, and inhibition of EHMT2 could reverse this phenotype [48], suggesting it may have a role in maintenance of the NE lineage, although this does not preclude an earlier role as well. Mutations in the KMT2-family of lysine methyltransferases are also common in pre-transformed NSCLC and T-SCLC [9,55], suggesting a potential role for these epigenetic factors in SCLC transformation. Additionally, EZH2 and other members of the PRC2 complex are also upregulated in T-SCLC [55]. Multiple studies have shown that inhibiting EZH2 does not reverse the NE phenotype of T-SCLC [48,55], suggesting it does not have a role in the maintenance of T-SCLC, although it is possible that it could have a role during early steps of transformation.

## 8. Prospects for T-SCLC Therapies

Patients with T-SCLC are typically treated with platinum/etoposide chemotherapy, the same chemotherapy backbone used for de novo SCLC [10,12,42]. Unfortunately, this does not provide long-term benefit, with most patients surviving less than one year after transformation [9,12,42]. This highlights the pressing need to identify novel therapeutic strategies for T-SCLC. Recent work has begun to uncover potential new therapies for T-SCLC (summarized in Table 1), although, to date, none have been approved. A recent study by Zang et al. found that combination chemotherapy plus immunotherapy substantially improved progression-free survival of T-SCLC compared to chemotherapy alone (~8 vs. ~20 months), suggesting this may be a better approach for T-SCLC management [42]. These combinatorial approaches are currently being tested in two ongoing phase 2 clinical trials (NCT05957510 [104] and NCT03944772 [105]). While combination chemotherapy with immunotherapy is now the standard for de novo SCLC, the survival benefit is much less than that found in the cohort of T-SCLC [42,106,107,108,109]. Whether these differences hold true in a larger cohort will be of great interest and would suggest a distinct difference between de novo and T-SCLC in regards to response to immunotherapy. It has been demonstrated that SCLC patients that exhibit an “inflamed” gene signature (e.g., SCLC-I) are more responsive to immunotherapy [110], so perhaps T-SCLC more closely resembles this inflamed subtype of SCLC. An ongoing phase 2 clinical trial (NCT04538378) is also testing the efficacy of immunotherapy in combination with PARP inhibition for T-SCLC. Despite the potential of these approaches, it is important to note that SCLC transformation represents a mechanism of resistance to immunotherapy in NSCLC [31,32,33,34,35,36,37,38,39], which would argue that these specific T-SCLC patients are unlikely to benefit from immunotherapy.

An alternative approach to treating T-SCLC would be to identify NSCLC patients at high risk of SCLC transformation and treat them proactively to prevent transformation. Identifying biomarkers that predict patients destined to undergo SCLC transformation will be critical for this approach. Thus far, the best marker of SCLC transformation is inactivation of *RB1* and *p53* in the pre-transformed NSCLC. An ongoing phase 1 clinical trial (NCT03567642) is testing the efficacy of combining the EGFR inhibitor, Osimertinib, with platinum/etoposide chemotherapy in patients with *EGFR*-mutant NSCLC that also possess *RB1* and *p53* mutations. Early data demonstrated the safety of the combined therapy, although it did not prevent SCLC transformation [111]. Whether this approach will improve patient survival requires further study. If this or other therapeutic approaches to prevent SCLC transformation are successful, it will be key to identify robust biomarkers of transformation. While *RB1* and *p53* inactivation are the best biomarkers to date, many NSCLC patients with *RB1/p53* loss do not undergo transformation [13]. Furthermore, emerging evidence suggests that SCLC transformation may be more common in settings outside of *EGFR*-mutant LUAD that previously thought [9,41]. Thus, our understanding as to the best markers to predict SCLC transformation requires improvement. Additional mutational signatures do not appear to predict transformation with greater efficacy than *RB1/p53* loss, but whether there are alternative transcriptomic or epigenetic signatures that do remains to be determined. Indeed, pre-transformed LUAD (that eventually undergoes SCLC transformation) exhibits a distinct transcriptional profile compared to LUAD that never undergoes transformation [55]. This suggests that it may be possible to define a robust transcriptional signature, perhaps in combination with *RB1/p53* inactivation, that accurately predicts SCLC transformation. Alternatively, it may be possible to closely monitor patients using non-invasive approaches to detect early stages of SCLC transformation. Recent work demonstrated the ability to accurately predict SCLC transformation in *EGFR*-mutant LUAD patients from cell-free DNA using a specific epigenetic signature [98], highlighting the exciting potential for this approach, although significant validation on diverse cohorts will be required to deploy this for clinical use.

Our improving knowledge of the molecular mechanisms underlying SCLC transformation may provide further therapeutic opportunities for T-SCLC. For example, studies by Quintanal-Villalonga et al. demonstrated that XPO1 [56] and CDC7 [57] play key roles in NE transformation, which highlighted the potential for targeting these proteins in T-SCLC. Indeed, inhibitors of XPO1 (Selinexor) [56] or CDC7 (Simurosertib/TAK-931) [57] showed some single agent efficacy against a mixed LUAD/SCLC PDX model and strong combinatorial effects with EGFR inhibition in this model. The authors also observed strong combinatorial effects when these inhibitors were combined with platinum/etoposide chemotherapy in PDX models derived from de novo as well as T-SCLC. The XPO1 inhibitor, Selinexor, is FDA approved for treatment of refractory multiple myeloma and is being tested in various other cancers [78], providing a potential path to testing in the context of T-SCLC. The CDC7 inhibitor, Simurosertib, has demonstrated safety in early clinical trials, and although it exhibited limited efficacy for several advanced solid tumors [112,113], specific approaches for transformed or de novo SCLC have yet to be explored. Combination therapies may represent the best approach for Simurosertib, as these approaches showed the best efficacy in SCLC PDX models [57]. Yang et al. also demonstrated the efficacy of pre-clinical EHMT2 inhibitors against T-SCLC cell line models [48], suggesting that targeting this axis may be a good approach for T-SCLC. Unfortunately, to date, EHMT2 inhibitors have not reached the clinical setting despite great interest in this area [114]. Thus, there are already several promising new approaches to explore clinically against T-SCLC, and as more is learned about the underlying etiology of this disease, it will surely unveil additional therapeutic strategies. Whether these approaches will be best utilized to target T-SCLC after transformation, or instead used to treat proactively to prevent transformation, will require further studies.

## 9. Future Perspectives

Since the initial descriptions of SCLC transformation as a mechanism of resistance to TKIs in *EGFR*-mutant NSCLC just under two decades ago [14,15,16,17,18], our understanding of this therapy-driven response has grown substantially. However, we still have much to learn, from fundamental aspects of SCLC conversion to detailed mechanistic understanding and, most importantly, how T-SCLC can be better treated. Surprisingly, our knowledge regarding the frequency of SCLC transformation beyond *EGFR*-mutant NSCLC is limited. Recent studies have shown that SCLC transformation may be an underappreciated phenomenon in *EGFR* wild-type NSCLC and could be more prevalent than in *EGFR*-mutant NSCLC [9,41]. It has also been speculated that the frequency of SCLC transformation will increase as new and improved targeted therapies are developed to target a wider range of oncogenic drivers [23,56]. Thus, it will be imperative to define the frequency of SCLC transformation in these different contexts, which will require a change in standard practices to include rebiopsy of tumors at the time of relapse or to adopt non-invasive techniques to identify SCLC transformation, such as cell-free DNA profiling [98].

In recent years, our understanding of the mechanisms that drive SCLC transformation has improved significantly; however, we have likely just scratched the surface of this understanding. For example, it is still unclear how heterogeneous the mechanisms are that drive SCLC transformation. Certain mutations that facilitate SCLC transformation, such as those in *SMAD4* [9,12,75], are found in only a fraction of patients, suggesting these may not have broad relevance. However, it is also possible that different events may converge on a small number of key pathways required to drive SCLC transformation. For example, *SMAD4* mutation [9], CDC7 upregulation [57] and *MYC* amplification [11,13,23,43] all function to promote the activity of MYC, which is a critical driver of SCLC transformation [50,53]. It will be interesting to better understand how these different pathways communicate, interconnect and converge to ultimately drive SCLC transformation. Furthermore, we know little about the timing of the different events that cooperate to drive transformation. While one can speculate on this (e.g., Figure 3), significant work is required to carefully map the temporal changes that occur during NSCLC-to-SCLC evolution and identify the mechanisms that act at different stages to drive these progressive changes.

While we have begun to understand the mechanisms underlying SCLC transformation, the specific cellular signals that initiate transformation are still unclear. While certain mutations predispose NSCLC to transformation (e.g., *RB1* and *p53* inactivation), these alone are not sufficient to drive transformation. Thus, there must be a specific signal(s) that initiates the process of SCLC transform. Perhaps this is a stress-sensing pathway triggered by therapeutic pressure or non-cell autonomous signals from tumor-associated stroma or dying tumor cells. Alternatively, stochastic mechanisms may be responsible, and a slight imbalance or change in cellular activity may be sufficient to tip the scales towards transformation. This would also align with the notion that certain tumors are primed to undergo transformation based on epigenetic or transcriptional profiles [55]. Although, why certain tumors are primed to undergo transformation is still unclear but could be influenced by distinct cells of origin, extra-cellular signals arising from unique tumor microenvironments, or non-coding mutations, among many other possibilities.

## 10. Conclusions

Our growing understanding of the mechanisms that drive SCLC transformation has unveiled potential strategies to predict, detect and treat T-SCLC, with several clinical trials arising from these early fundamental discoveries. These opportunities will only grow as we better understand the underlying biology of SCLC transformation. The ability to more accurately detect cases of T-SCLC, particularly early in the disease course, will be critical to improving patient outcomes, but better yet would be to identify biomarkers to predict patients destined to undergo SCLC transformation. To date, the best indicator of this is inactivation of *RB1* and *p53* in the pre-transformed NSCLC, which increases the risk of transformation by 6-fold [13]. However, the majority of *RB1/p53*-null patients do not undergo transformation [13]. Thus, it will be critical to identify better biomarkers that predict transformation, potentially through transcriptional or epigenetic signatures. Even when these patients can be more accurately identified, current treatments for T-SCLC are largely ineffective, so it will be essential to identify better treatment strategies to target T-SCLC. Ultimately, these discoveries will be driven by a better understanding of the mechanisms that underlie SCLC transformation, although these approaches will likely be complicated by heterogeneity in the mechanisms of SCLC transformation. While we have come a long way in our understanding of T-SCLC in recent years, including identifying potential therapeutic strategies and biomarkers, we still have much to learn about the mechanisms that drive SCLC transformation and how these can be exploited therapeutically.

## Figures and Tables

**Table 1:** Summary of approved, emerging and potential therapies for T-SCLC.

Therapeutic Approach	Drugs	Stage of Development	Treatment Phase	Reference
Standard chemotherapy	Platinum/ etoposide	Approved	T-SCLC	
Chemotherapy + immunotherapy	Serplulimab + platinum/ etoposide	Ongoing Phase 2 trials	T-SCLC	NCT05957510, NCT03944772
Immunotherapy + PARP inhibition	Olaparib + Durvalumab	Ongoing Phase 2 trial	T-SCLC	NCT04538378
EGFR inhibitor + chemotherapy	Osimertinib + platinum/ etoposide	Ongoing Phase 1 trial	NSCLC with EGFR/RB/TP53 alterations	NCT03567642
XPO1 inhibition	Selinexor	Preclinical evidence	Not applicable	[56]
CDC7 inhibition	Simurosertib/ TAK-931	Preclinical evidence	Not applicable	[57]
EHMT2 inhibition	UNC0638, UNC0642	Preclinical evidence	Not applicable	[48]
WNT/β-catenin inhibition	MSAB	Preclinical evidence	Not applicable	[48]
